# Reviewing the epidemiology of head and neck cancer: definitions, trends and risk factors

**DOI:** 10.1038/s41415-022-5166-x

**Published:** 2022-11-11

**Authors:** Mark Gormley, Grant Creaney, Andrew Schache, Kate Ingarfield, David I. Conway

**Affiliations:** 4141578122001 Consultant Senior Lecturer in Oral Surgery, Medical Research Council, Integrative Epidemiology Unit, Population Health Sciences, Bristol Medical School, University of Bristol, UK; 4141578122002 Clinical Lecturer in Dental Public Health, School of Medicine, Dentistry and Nursing, University of Glasgow, Glasgow, UK; 4141578122003 Reader in Head and Neck Surgery and Honorary Consultant Head and Neck Surgeon, Institute of Translational Medicine, University of Liverpool, UK; 4141578122004 Research Associate Centre for Trials Research, Cardiff University, UK; 4141578122005 Professor of Dental Public Health, School of Medicine, Dentistry and Nursing, University of Glasgow, UK

## Abstract

**Introduction ** Head and neck cancer appears to be increasing in incidence, with potential changes in aetiology proposed. This paper aims to provide a narrative overview of the epidemiological literature to describe the disease burden and trends in terms of incidence and mortality both in the UK and globally and to review the evidence on current risk factors.

**Methods**A search was performed on multiple databases (PubMed and Epistemonikos), applying filters to identify systematic reviews and meta-analyses which investigated head and neck cancer incidence, mortality and risk factors. International and UK cancer registries and sources were searched for incidence and mortality data.

**Results ** Multiple definitions of head and neck cancer are employed in epidemiology. Globally, incidence rates have increased in recent decades, largely driven by oropharyngeal cancer. Mortality rates over the last decade have also started to rise, reflecting the disease incidence and static survival rates. Major risk factors include tobacco smoking alone and in combination with alcohol consumption, betel chewing (particularly in Southeast Asian populations) and the human papillomavirus in oropharyngeal cancer.

**Conclusions**These epidemiological data can inform clinical and preventive service planning for head and neck cancer.

## Key points

- Head and neck cancer incidence is increasing and is projected to continue to rise, largely driven by increases in oropharyngeal cancer.
- Mortality rates have started to increase within the last decade, reflecting a rising incidence and static survival rates.
- Major risk factors that are associated with the risk of head and neck cancer are tobacco smoking and tobacco used in combination with alcohol consumption. Human papillomavirus is an additional major risk factor for oropharyngeal cancer. Cancers of the head and neck are clearly socioeconomically patterned but this socioeconomic risk is not entirely explained by smoking and alcohol behaviours.

## Introduction

Head and neck cancer (HNC) is the seventh most common cancer globally, accounting for more than 660,000 new cases and 325,000 deaths annually.^1^^,^^2^ There appears to be an increasing incidence of this disease, with potential changes in aetiology proposed given the decline of smoking, particularly in developed countries.^3^ Epidemiology is both descriptive - describing the burden and trends of disease - and analytical - identifying risk factors. This paper aims to provide a narrative overview of the epidemiological literature, to describe the disease burden and trends in terms of incidence and mortality both in the UK and globally, and to review the evidence on current risk factors.

## Definitions of head and neck cancer

Approximately 90% of HNCs are squamous cell carcinoma, which arise from the epithelial lining of the oral cavity, pharynx and larynx.^1^ There are many types of cancers affecting the head and neck, which are discretely categorised on the basis of their anatomical location using the International Classification of Diseases (ICD-10) from the World Health Organisation (WHO).^2^ A list of each subsite (including the individual ICD-10 code) is outlined in Table 1. Due to the differences in the presenting symptoms, treatment regimens and prognosis at each anatomical subsite, these are considered as separate entities.^3^ A comprehensive review carried out by Kaste *et al*.^4^ outlined the several differences in the contributory elements to the definition of HNC from major institutions such as the National Cancer Institute, International Agency for Research on Cancer (IARC) and Cancer Research UK (CRUK). The most important difference between these definitions is the inclusion of the oesophagus under the definition set out by CRUK. Several epidemiological studies include this subsite as a form of HNC, or under an overarching upper aerodigestive tract cancer definition. While some clinical definitions also include thyroid cancers, these are usually excluded in epidemiological studies. Due to variations in definition, it is important anatomical subsites are clearly specified when reviewing the literature (ideally by using corresponding ICD codes. See Table 1).

**Table 1:** Anatomical subsites of the head and neck based on ICD-10

Main site	ICD-10 code
Malignant neoplasms of lip	C00
Malignant neoplasm of base of tongue	C01
Malignant neoplasm of other and unspecified part of tongue	C02
Malignant neoplasm of gum	C03
Malignant neoplasm of floor of mouth	C04
Malignant neoplasm of palate	C05
Malignant neoplasm of other and unspecified parts of mouth	C06
Malignant neoplasm of parotid gland	C07
Malignant neoplasm of other and unspecified major salivary glands	C08
Malignant neoplasm of tonsil	C09
Malignant neoplasm of oropharynx	C10
Malignant neoplasm of nasopharynx	C11
Malignant neoplasm of piriform sinus	C12
Malignant neoplasm of hypopharynx	C13
Malignant neoplasm of other and ill-defined sites in the lip, oral cavity and pharynx	C14
Malignant neoplasm of nasal cavity and middle ear	C30
Malignant neoplasm of accessory sinuses	C31
Malignant neoplasm of larynx	C32
Malignant neoplasm of other and ill-defined sites	C76

## Methods

A search was performed on multiple databases (PubMed and Epistemonikos), applying filters to identify systematic reviews and meta-analyses which investigated HNC incidence, mortality and risk factors, using the IARC definition and corresponding ICD-10 codes described in Table 1. International and UK cancer registries and sources were searched for incidence and mortality data.

The International Head and Neck Cancer Epidemiology (INHANCE) consortium has performed the largest pooled analyses using individual level data from studies from around the world, with the overall aim to better understand the aetiology and risks associated with HNC. These analyses have the advantage of harmonising definitions of disease outcomes, definition of risk factors and the ability to obtain robust estimates, adjusting for confounding and other potential effect modifiers. The INHANCE methods have been described in detail elsewhere and further information is available on the INHANCE consortium website, hosted by the University of Utah.^5^ Briefly, there are 35 pooled case-control studies with a total of over 25,700 HNC cases and 37,100 controls. Included studies have minimal criteria on sample size and data availability and are usually multicentre studies, with 15 from North America, 13 from Europe, 3 from Latin America, 3 from Asia and 1 from multiple continents. There are important limitations of these INHANCE analyses which have been acknowledged, mainly related to inherent drawbacks in the original case-control source studies and despite the greatest global burden of disease being in Southeast Asia, there are few studies included from this region (with no studies from India and Bangladesh) or from Africa. INHANCE have published over 45 peer-reviewed papers, including analyses on the following HNC risks: tobacco smoking, alcohol drinking, diet/anthropometrics, oral health/hygiene, medical history, sexual history, genetics, occupation and socioeconomics. All of the INHANCE results to date have been summarised in two overview papers^6^^,^^7^ and key findings will be summarised here.

## Results

Understanding the cancer burden is key to developing, managing and improving services for disease prevention and treatment. Here, peer-reviewed literature and cancer registry data are used to describe the latest incidence, mortality and risk factor trends for HNC.

### Global incidence trends in head and neck cancer

**Fig. 1:**
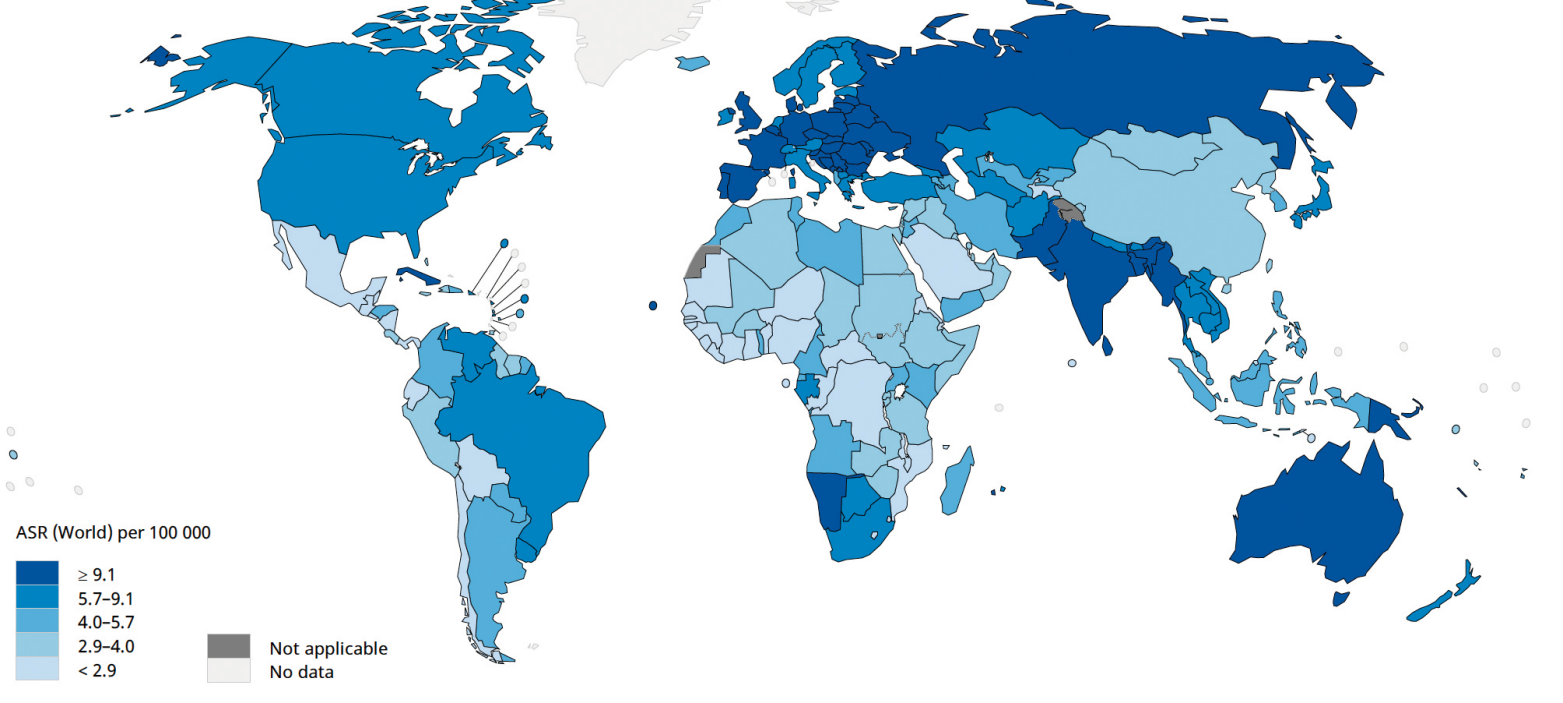
Global age-standardised incidence rates of head and neck cancer. Reprinted from International Agency For Research On Cancer, 'Cancer today - Data visualization tools for exploring the global cancer burden in 2020', Copyright 2022, http://gco.iarc.fr/today (accessed August 2021). The map was generated using the GLOBOCAN website mapping tool (https://gco.iarc.fr/today/online-analysis-map) by selecting the 'lip, oral cavity', 'oropharynx', hypopharynx' and 'larynx' cancer sites. Estimated age-standardised rates of head and neck cancer incidence worldwide are shown for both sexes

The overall incidence of HNC continues to rise, with a predicted 30% increase annually by 2030.^1^^,^^2^ This increase has been recorded across both developed and developing countries.^6^ Southeast Asia and Asia-Pacific regions have a particularly high incidence of oral cancer, associated with chewing of areca nut (betel quid), with or without tobacco.^8^ Oral cancer is therefore expected to rise within Southeast Asia, in line with population growth.^9^ The increasing incidence rates of HNC in the USA and Europe have been attributed to a rise in oropharyngeal cancer, linked to human papillomavirus (HPV) infection^10^^,^^11^ (Fig. 1). Recent studies have demonstrated a global trend towards increasing incidence in HPV-related subsites, accompanied by decreasing incidence in HPV-unrelated subsites in countries such as the USA, Canada, Hong Kong and Korea.^12^ Over the next 20 years, it is expected that the majority of HNC will be HPV-positive, with projections that in some European countries, such as the UK, oropharyngeal cancer incidence will overtake cancer of the oral cavity.^13^

Worldwide, laryngeal cancers have increased by 23% over the past ten years. However, age-adjusted rates for new laryngeal cancer cases have been falling in countries with a higher sociodemographic index, again reflecting changes in smoking and alcohol drinking behaviour.^14^ Overall, HNC affects men two to four times more than women, with estimates reaching over 20 per 100,000.^15^ For men in developing countries, lip and oral cavity cancer is the second most common cancer (10 per 100,000). Male incidence of oral and oropharyngeal cancer has declined over recent years in France (-12.6%), Slovakia (-4.0%), Spain (-10.8%), Brazil (-26.7%) and Hong Kong (-10.5%), while it increased in the UK (18.8%), Australia (8.7%), Japan (21.3%) and in the US (3.7%).^16^ The risk of HNC increases with age across populations, with the majority of cases diagnosed in those over 50 years.^17^ Similarly, there has been a rise in cases among younger women, predominantly in European countries, which may be explained by sex-specific patterns of tobacco and alcohol consumption.^15^^,^^16^

### The incidence of head and neck cancer in the UK

HNC rates are also rising in the UK. Studies have shown that from 1995-2011, oropharyngeal cancer incidence increased by 7.3% for men and 6.5% for women in England, with oral cavity cancer showing a 2.8% rise in men and 3.0% rise in women over the same period.^18^ Incidence rates are highest in Scotland, where oropharyngeal cases were shown to have increased by 85% from 2001-2012.^19^ These increasing rates are continuing to rise according to the most-recent UK Cancer Registry data, which show a 34% increase in total cases diagnosed across the four nations from 2011-2018. The burden of HNC is strongly socioeconomically patterned, with the highest rates being observed among people living in the most socioeconomically deprived communities.^19^ These Cancer Registry data also demonstrate that the majority of HNCs are diagnosed at an advanced stage.^20^ For the UK as a whole, 58.5% of HNCs are diagnosed at stage III or IV, in accordance with the seventh edition of the TNM (tumor-node-metastasis) Atlas.^21^ Overall, stage IV is the most common stage at diagnosis for oral and oropharyngeal cancer, whereas stage I is most common for laryngeal cancer.^20^

### Mortality and survival trends in head and neck cancer

In 2018, there were 4,078 deaths attributable to HNC in the UK, accounting for approximately 2% of all cancer deaths annually.^22^ There is apparent variation in national HNC mortality rates (European age-standardised rates [ASR]; ASR per 100,000 population) between nations of the UK. Scotland (ASR 8.7) and Northern Ireland (ASR 8.4) had worse outcomes than England (ASR 6.2) and Wales (ASR 5.8). Age-specific mortality that is attributable to HNC rises from the fifth decade onwards, towards a peak mortality in those over the age of 90 years; a phenomenon most pronounced in men. In the UK, since the early 1970s, the combined HNC mortality for men and women has fallen by 11% overall (1971: ASR 7.3; 2018: ASR 6.5); however, the last decade has seen a gradual rise in mortality rates from a low in 2006 (ASR 5.6), possibly reflecting the changes in disease incidence and static survival rates.^22^

Globally, five-year survival for HNC averages at 50% of cases, with hypopharynx experiencing the worst outcomes.^17^ A recent analysis of WHO death certification data showed that there has been little change in mortality generally over recent decades for both men and women.^16^ Survival rates can vary significantly across geographical location, tumour site and, most prominently, stage at diagnosis. Those diagnosed with advanced disease have notably poorer outcomes than those with early disease. Analysis of a large cohort in the USA showed that HPV-positive cancers had a better chance of long-term survival compared to non-HPV cancers, confirming previous studies also suggesting this phenomenon.^23^ There are several studies exploring the factors associated with relatively poor survival among people with HNC, including Head and Neck 5000^24^ and the recently established HEADSpAcE.^25^ Analysis of routinely collected data and from large prospective cohort studies, such as Head and Neck 5000, have demonstrated that significant socioeconomic inequalities in HNC survival exist in the UK, not all of which can be explained by behavioural factors.^26^

### Risk factors associated with head and neck cancer

Tobacco smoking and alcohol drinking behaviours, separately and in combination, are major risk factors for HNC, accounting for 72% of cases when used in combination.^27^ However, this was slightly less for oral cavity cancer (64%) and slightly more for laryngeal cancer (89%) and much lower for women and among younger age groups.^27^ Risk factors are generally similar across subsites of the head and neck, although the magnitude of risk associated with particular risks may vary, for example tobacco smoking risks are greatest for laryngeal cancer and alcohol for oral cavity and oropharyngeal cancer.^28^ In a large pooled analysis, Hashibe *et al*. investigated the independent effects of tobacco smoking in never alcohol drinkers and found an increased odds risk (odds ratio [OR] 2.13; 95% confidence internal [CI] = 1.53, 2.98) of HNC,^29^ with a further study examining interaction suggesting a multiplicative joint effect of tobacco and alcohol (Table 2).^27^ More recent studies have demonstrated evidence for an independent causal effect of alcohol consumption in oral and oropharyngeal cancer when controlling for smoking, suggesting the role of alcohol may have been previously underestimated.^30^ Dietary^31^ and other lifestyle factors, such as physical activity^32^ and oral hygiene,^33^ are currently considered relatively minor risk factors (Table 2).

**Table 2:** Risk factors for head and neck cancer

Risk factor for HNC (unless specified)	Level of exposure	Range of OR or population attributable risk (PAR) (95% CI)	Reference
Tobacco smoking in never-alcohol drinkers	Cigarette smoking vs never	OR 2.13 (1.53, 2.98)	Hashibe *et al*., 2007^29^
Tobacco smoking by type of product	Cigarettes (+ dose response)	OR 3.46 (3.24, 3.70)	Wyss *et al*., 2013^47^
	Cigars (+ dose response)	OR 2.54 (1.93, 3.34)	
	Pipes (+ dose response)	OR 2.08 (1.55, 2.81)	
Passive smoking	Home vs never	OR 1.60 (1.12, 2.28)	Lee *et al*., 2008^48^
	Work vs never	OR 1.55 (1.04, 2.30)	
Smokeless tobacco	Snuff vs never	OR 1.71 (1.08, 2.70)	Wyss *et al*., 2016^49^
	Chewing tobacco vs never	OR 1.20 (0.81, 1.77)	
Smokeless tobacco (oral cancer)	Gutka vs never	OR 8.67 (3.59, 20.93)*	Asthana *et al*., 2019^50^
	Pan/areca nut/lime/betel liquid	OR 7.18 (5.48, 9.41)*	
	Manipuri vs never	OR 3.32 (1.32, 8.36)*	
Alcohol in never users of tobacco	Three or more drinks per day vs never	OR 2.04 (1.29, 3.21)	Hashibe *et al*., 2007^29^
Alcohol beverage type	Wine >30 drinks/week vs never	OR 6.3 (2.2, 18.6)	Purdue *et al*., 2009^51^
	Beer >30 drinks/week vs never	OR 5.4 (3.1, 9.2)	
	Liquor >30 drinks/week vs never	OR 3.6 (2.2, 5.8)	
Combined effects of alcohol and drinking	Never users	OR 1.00 (Ref.)	Hashibe *et al*., 2009^27^
	Tobacco alone	OR 2.37 (1.66, 3.39)	
		PAR 33.0 (42.6, 25.9)	
	Alcohol alone	OR 1.06 (0.88, 1.28)	
		PAR 4.0 (1.5, 5.3)	
	Tobacco and alcohol joint effect	OR 5.73 (3.62, 9.06)	
		PAR 34.9 (17.2, 48.0)	
	Total	PAR 72.0 (61.2, 79.1)	
Racial differences in alcohol risk	Never to <20 years	OR 1.00 (Ref.)	Voltzke *et al*., 2018^52^
	≥20 to 30 years	White 1.62 (1.36, 1.94)	
		Black 2.01 (1.07, 3.79)	
	≤30 years	White 1.38 (1.20, 1.58)	
		Black 2.20 (1.38, 3.50)	
Marijuana	Ever use vs never	OR 0.88 (0.67, 1.16)	Berthiller *et al*., 2009^53^
Diet - consumption of food items/types	High fruit vs low	OR 0.52 (0.43, 0.62)	Chuang *et al*., 2012^31^
	High vegetables vs low	OR 0.66 (0.49, 0.90)	
	High red meat vs low	OR 1.40 (1.13, 1.74)	
	High processed meat vs low	OR 1.37 (1.14, 1.65)	
	Low (<18.5) vs normal	OR 1.69 (1.10, 2.50)	
BMI (larynx)	Overweight/obese vs normal	OR 0.60 (0.3, 1.3)	Lubin *et al*., 2011^28^
Recreational physical activity	Moderate vs none/low	OR 0.78 (0.66, 0.91)	Nicolotti *et al*., 2011^32^
Diabetes	History of diabetes vs no history	OR 1.09 (0.95, 1.24)	Stott-Miller *et al*., 2012^54^
Sexual behaviours (oropharyngeal cancer)	≥6 lifetime sexual partners vs 1	OR 1.25 (1.01, 1.54)	Heck *et al*., 2010^35^
	≥4 lifetime oral sex partners vs 0-1	OR 2.25 (1.42, 3.58)	
HPV	Oropharynx tonsil HPV16 vs negative	OR 15.1 (6.8, 33.7)	Hobbs *et al*., 2006^34^
	Oropharynx other HPV16 vs negative	OR 4.3 (2.1, 8.9)	
	Oral cavity HPV16 vs negative	OR 2.0 (1.2, 3.4)	
	Larynx HPV16 vs negative	OR 2.0 (1.0, 4.2)	
Oral health/hygiene	<5 missing teeth vs ≥missing teeth	OR 0.78 (0.74, 0.82)	Hashim *et al*., 2016^33^
	No gum disease vs gum disease	OR 0.94 (0.89, 0.99)	
	Annual dentist vs <once a year	OR 0.82 (0.78, 0.87)	
	Daily toothbrushing vs <once a day	OR 0.83 (0.79, 0.88)	
Family history	First degree relative with HNC	OR 1.7 (1.2, 2.3)	Negri *et al*., 2009^55^
Increased duration in occupations	Cooks	OR 1.36 (1.09, 1.68)	Khetan *et al*., 2019^56^
	Cleaners	OR 1.38 (1.13, 1.69)	
	Painters	OR 1.82 (1.42, 2.35)	
Socioeconomic factors	Low vs high education	OR 2.50 (2.02, 3.09)	Conway *et al*., 2015^57^
	Low vs high income	OR 2.44 (1.62, 3.67)	
	Low vs high occupational socioeconomic status	OR 1.88 (1.64, 2.17)	Conway *et al*., 2021^58^
Key: * = Risk estimates adjusted for confounders unless indicated			

High-risk HPV, especially HPV type 16, is a major risk factor for oropharyngeal cancer,^34^ thought to be sexually transmitted via oro-genital contact (Table 2).^11^ Heck *et al*. demonstrated that >4 oral sex partners greatly increased the risk of oropharyngeal cancer (OR 2.25; 95% CI = 1.42, 3.58).^35^ HPV has been associated with over 50% of cases in the UK^36^ but there is wide global variation^37^^,^^38^ and smoking has been shown to interact with HPV and increase risk.^39^ Those who have HPV-negative oropharyngeal tumours are more likely to be heavier smokers, with an increased risk of death for every additional pack-year, compared to HPV-positive cases.^40^ HPV-positive cases present with an almost 60% reduction in the risk of mortality after adjustment for prognostic factors, such as age, ethnicity, staging, smoking status and treatment regime.^40^ This could be because people with HPV-positive disease are slightly younger, with less comorbidity, or that they have enhanced anti-tumour immunity. It has also been postulated that HPV-positive tumours harbour fewer genetic mutations or may be more radiosensitive (with an intact apoptotic response), associated with an overall better response to radiotherapy.^41^

Genetic susceptibility to HNC has been investigated,^42^ with the largest genome-wide association study (GWAS) of oral and pharyngeal cancer (6,034 cases and 6,585 controls from Europe, North America and South America), detecting seven unique loci.^43^ Of note, oropharyngeal subgroup analysis revealed a strong protective association at chromosome 6p21.32 (lead variant rs3828805, mapping to HLA-DQB1) within the human leucocyte antigen (HLA) Class II region. A more recent GWAS in an independent dataset supports these findings.^44^ Follow-up is required to determine the extent and specificity of the HLA effect in HPV-positive tumours, which could help explain why some individuals are more at risk of developing the disease following HPV infection. Genetic variants in alcohol-metabolising genes, such as alcohol dehydrogenase, have also been associated with increased HNC risk.^45^^,^^46^

Tobacco smoking and alcohol consumption are established risk factors for HNC.^27^ However, a detailed understanding of these somewhat complex behaviours in terms of precise estimates of risk, understanding the joint tobacco-alcohol effect, the dose-response, and the benefits of quitting both smoking and alcohol, remain less well established. The role of other potential risk factors, such as smokeless tobacco, betel chewing, diet, oral health and hygiene and hormonal, genetic, occupational and socioeconomic status in HNC risk, are not well understood. A major challenge in elucidating detailed information from the epidemiological literature is the heterogeneity in study designs and populations often being from small observational studies. The dominant effect of tobacco smoking and alcohol drinking also overshadow other potential risk factors. While a number of the systematic reviews and meta-analyses presented aggregated estimates from epidemiological studies, these were usually focused on specific risk factors and/or subsites of the head and neck. Furthermore, these studies often encountered difficulties harmonising data from observational studies and adjusting for appropriate confounding factors. Observational studies also suffer from other methodological issues, such as reverse causality. Given that multiple HNC definitions were used, this adds complexity to the interpretation of results.

## Conclusions

Both globally and in the UK, HNC incidence has increased over recent decades and is projected to continue to rise, largely driven by increases in oropharyngeal cancer. Mortality rates in the UK have started to increase within the last decade, reflecting rising incidence and static survival rates. In summary, the major risk factors that are associated with the risk of HNC are tobacco smoking and tobacco used in combination with alcohol consumption. Betel chewing is an established risk in Southeast Asian countries and in people from Southeast Asian minority ethnic groups. HPV is an additional major risk factor for oropharyngeal cancer. Cancers of the head and neck are clearly socioeconomically patterned, with those from the poorest backgrounds having the greatest burden, but this socioeconomic risk is not entirely explained by smoking and alcohol behaviours. Moreover, HNCs are higher among men than women (although the trends are diverging) and more common in older age-groups, although oropharyngeal cancer incidence peaks around ten years younger, at around 60-65 years.

## References

[1] Sung H, Ferlay J, Siegel R L *et al.* Global Cancer Statistics 2020: GLOBOCAN Estimates of Incidence and Mortality Worldwide for 36 Cancers in 185 Countries. *CA Cancer J Clin* 2021; **71:** 209-249. 10.3322/caac.21660 33538338

[2] Johnson D E, Burtness B, Leemans C R, Lui V W Y, Bauman J E, Grandis J R. Head and neck squamous cell carcinoma. *Nat Rev Dis Primers* 2020; **6:** 92. 10.1038/s41572-020-00224-3 PMC7944998 33243986

[3] Thomas S J, Penfold C M, Waylen A, Ness A R. The changing aetiology of head and neck squamous cell cancer: A tale of three cancers? *Clin Otolaryngol* 2018; **43:** 999-1003. 10.1111/coa.13144 29770611

[4] Kaste L, Dolecek T A, Zavras A I. Head and Neck Cancer Epidemiology and Health Services Research. *In* Radosevich J A (ed) *Head & Neck Cancer: Current Perspectives, Advances, and Challenges*. Chicago, IL: Springer Dordrecht Heidelberg New York London, 2013.

[5] The International Head and Neck Cancer Epidemiology (INHANCE) Consortium. About Us. 2004. Available at https://medicine.utah.edu/dfpm/inhance (accessed October 2022).

[6] Bravi F, Lee Y-C A, Hashibe M *et al.* Lessons learned from the INHANCE consortium: An overview of recent results on head and neck cancer. *Oral Dis* 2021; **27:** 73-93. 10.1111/odi.13502 PMC7752834 32569410

[7] Winn D, Lee Y-C, Hashibe M, Boffetta P, INHANCE consortium. The INHANCE consortium: toward a better understanding of the causes and mechanisms of head and neck cancer. *Oral Dis* 2015; **21:** 685-693. 10.1111/odi.12342 25809224

[8] Shield K D, Ferlay J, Jemal A *et al.* The global incidence of lip, oral cavity, and pharyngeal cancers by subsite in 2012. *CA Cancer J Clin* 2017; **67:** 51-64. 10.3322/caac.21384 28076666

[9] Cheong S C, Vatanasapt P, Yi-Hsin Y, Zain R B, Kerr A R, Johnson N W. Oral cancer in South East Asia: Current status and future directions. *Transl Res Oral Oncol* 2017; DOI: 10.1177/2057178X17702921.

[10] Mehanna H, Beech T, Nicholson T *et al.* Prevalence of human papillomavirus in oropharyngeal and nonoropharyngeal head and neck cancer-systematic review and meta-analysis of trends by time and region. *Head Neck* 2013; **35:** 747-755. 10.1002/hed.22015 22267298

[11] Gillison M L, Chaturvedi A K, Anderson W F, Fakhry C. Epidemiology of Human Papillomavirus-Positive Head and Neck Squamous Cell Carcinoma. *J Clin Oncol* 2015; **33:** 3235-3242. 10.1200/JCO.2015.61.6995 PMC4979086 26351338

[12] Menezes F D S, Fernandes G A, Antunes J L F, Villa L L, Toporcov T N. Global incidence trends in head and neck cancer for HPV-related and-unrelated subsites: A systematic review of population-based studies. *Oral Oncol* 2021; **115:** 105177. 10.1016/j.oraloncology.2020.105177 33561611

[13] Conway D I, Purkayastha, M, Chestnutt I G. The changing epidemiology of oral cancer: definitions, trends, and risk factors. *Br Dent J* 2018; **225:** 867-873. 10.1038/sj.bdj.2018.922 30412558

[14] Global Burden of Disease Cancer Collaboration. Global, Regional, and National Cancer Incidence, Mortality, Years of Life Lost, Years Lived With Disability, and Disability-Adjusted Life-years for 32 Cancer Groups, 1990 to 2015: A Systematic Analysis for the Global Burden of Disease Study. *JAMA Oncol* 2017; **3:** 524-548. 10.1001/jamaoncol.2016.5688 PMC6103527 27918777

[15] Miranda-Filho A, Bray F. Global patterns and trends in cancers of the lip, tongue and mouth. *Oral Oncol* 2020; **102:** 104551. 10.1016/j.oraloncology.2019.104551 31986342

[16] Bosetti C, Carioli G, Santucci C *et al.* Global trends in oral and pharyngeal cancer incidence and mortality. *Int J Cancer* 2020; **147:** 1040-1049. 10.1002/ijc.32871 31953840

[17] Warnakulasuriya S. Global epidemiology of oral and oropharyngeal cancer. *Oral Oncol* 2009; **45:** 309-316. 10.1016/j.oraloncology.2008.06.002 18804401

[18] Louie K S, Mehanna H, Sasieni P. Trends in head and neck cancers in England from 1995 to 2011 and projections up to 2025. *Oral Oncol* 2015; **51:** 341-348. 10.1016/j.oraloncology.2015.01.002 25619734

[19] Purkayastha M, McMahon A D, Gibson J, Conway D I. Trends of oral cavity, oropharyngeal and laryngeal cancer incidence in Scotland (1975-2012) - A socioeconomic perspective. *Oral Oncol* 2016; **61:** 70-75. 10.1016/j.oraloncology.2016.08.015 27688107

[20] CancerData. Staging data in England. Available at https://www.cancerdata.nhs.uk/stage_at_diagnosis (accessed June 2022).

[21] Brierley J, Asamura H, van Eycken E, Rous B. *TNM Atlas.* 7th ed. New Jersey: Wiley-Blackwell, 2021.

[22] Cancer Research UK. Head and neck cancers mortality statistics. Available at https://www.cancerresearchuk.org/health-professional/cancer-statistics/statistics-by-cancer-type/head-and-neck-cancers/mortality (accessed October 2022).

[23] Du E, Mazul A L, Farquhar D *et al.* Long-term Survival in Head and Neck Cancer: Impact of Site, Stage, Smoking, and Human Papillomavirus Status. *Laryngoscope* 2019; **129:** 2506-2513. 10.1002/lary.27807 PMC6907689 30637762

[24] Ness A R, Waylen A, Hurley K *et al.* Establishing a large prospective clinical cohort in people with head and neck cancer as a biomedical resource: head and neck 5000. *BMC Cancer* 2014; **14:** 973. 10.1186/1471-2407-14-973 PMC4301458 25519023

[25] International Agency for Research on Cancer. HEADSpAcE. 2022. Available at https://headspace.iarc.fr/ (accessed October 2022).

[26] Ingarfield K, McMahon A D, Hurley K *et al.* Inequality in survival of people with head and neck cancer: Head and Neck 5000 cohort study. *Head Neck* 2021; **43:** 1252-1270. 10.1002/hed.26589 33415733

[27] Hashibe M, Brennan P, Chuang S-C *et al.* Interaction between tobacco and alcohol use and the risk of head and neck cancer: pooled analysis in the International Head and Neck Cancer Epidemiology Consortium. *Cancer Epidemiol Biomarkers Prev *2009; **18:** 541-550. 10.1158/1055-9965.EPI-08-0347 PMC3051410 19190158

[28] Lubin J H, Muscat J, Gaudet M M *et al.* An examination of male and female odds ratios by BMI, cigarette smoking, and alcohol consumption for cancers of the oral cavity, pharynx, and larynx in pooled data from 15 case-control studies. *Cancer Causes Control* 2011; **22:** 1217-1231. 10.1007/s10552-011-9792-x PMC3304584 21744095

[29] Hashibe M, Brennan P, Benhamou S *et al.* Alcohol drinking in never users of tobacco, cigarette smoking in never drinkers, and the risk of head and neck cancer: pooled analysis in the International Head and Neck Cancer Epidemiology Consortium. *J Natl Cancer Inst* 2007; **99:** 777-789. 10.1093/jnci/djk179 17505073

[30] Gormley M, Dudding T, Sanderson E *et al.* A multivariable Mendelian randomization analysis investigating smoking and alcohol consumption in oral and oropharyngeal cancer. *Nat Commun* 2020; **11:** 6071. 10.1038/s41467-020-19822-6 PMC7695733 33247085

[31] Chuang S-C, Jenab M, Heck J E *et al.* Diet and the risk of head and neck cancer: a pooled analysis in the INHANCE consortium. *Cancer Causes Control* 2012; **23:** 69-88. 10.1007/s10552-011-9857-x PMC3654401 22037906

[32] Nicolotti N, Chuang S-C, Cadoni G *et al.* Recreational physical activity and risk of head and neck cancer: a pooled analysis within the international head and neck cancer epidemiology (INHANCE) Consortium. *Eur J Epidemiol *2011; **26:** 619-628. 10.1007/s10654-011-9612-3 21842237

[33] Hashim D, Sartori S, Brennan P *et al.* The role of oral hygiene in head and neck cancer: results from International Head and Neck Cancer Epidemiology (INHANCE) consortium. *Ann Oncol *2016; **27:** 1619-1625. 10.1093/annonc/mdw224 PMC4959929 27234641

[34] Hobbs C G L, Sterne J A C, Bailey M, Heyderman R S, Birchall M A, Thomas S J. Human papillomavirus and head and neck cancer: a systematic review and meta-analysis. *Clin Otolaryngol* 2006; **31:** 259-266. 10.1111/j.1749-4486.2006.01246.x 16911640

[35] Heck J E, Berthiller J, Vaccarella S *et al.* Sexual behaviours and the risk of head and neck cancers: a pooled analysis in the International Head and Neck Cancer Epidemiology (INHANCE) consortium. *Int J Epidemiol* 2010; **39:** 166-181. 10.1093/ije/dyp350 PMC2817092 20022926

[36] Schache A G, Powell N G, Cuschieri K S *et al.* HPV-Related Oropharynx Cancer in the United Kingdom: An Evolution in the Understanding of Disease Etiology. *Cancer Res* 2016; **76:** 6598-6606. 10.1158/0008-5472.CAN-16-0633 PMC9158514 27569214

[37] Mehanna H, Franklin N, Compton N *et al.* Geographic variation in human papillomavirus-related oropharyngeal cancer: Data from 4 multinational randomized trials. *Head Neck* 2016; DOI: 10.1002/hed.24336. 10.1002/hed.24336 PMC4869674 26749143

[38] Anantharaman D, Abedi-Ardekani B, Beachler D C *et al.* Geographic heterogeneity in the prevalence of human papillomavirus in head and neck cancer. *Int J Cancer* 2017; **140:** 1968-1975. 10.1002/ijc.30608 PMC8969079 28108990

[39] Anantharaman D, Muller D C, Lagiou P *et al.* Combined effects of smoking and HPV16 in oropharyngeal cancer. *Int J Epidemiol* 2016; **45:** 752-761. 10.1093/ije/dyw069 PMC5841602 27197530

[40] Ang K K, Harris J, Wheeler R *et al.* Human papillomavirus and survival of patients with oropharyngeal cancer. *N Engl J Med* 2010; **363:** 24-35. 10.1056/NEJMoa0912217 PMC2943767 20530316

[41] Elrefaey S, Massaro M A, Chiocca S, Chiesa F, Ansarin M. HPV in oropharyngeal cancer: the basics to know in clinical practice. *Acta Otorhinolaryngol Ital* 2014; **34:** 299-309. PMC4299160 25709145

[42] Vukovic V, Stojanovic J, Vecchioni A, Pastorino R, Boccia S. Systematic Review and Meta-analysis of SNPs from Genome-Wide Association Studies of Head and Neck Cancer. *Otolaryngol Head Neck Surg* 2018; **159:** 615-624. 10.1177/0194599818792262 30126334

[43] Lesseur C, Diergaarde B, Olshan A F *et al.* Genome-wide association analyses identify new susceptibility loci for oral cavity and pharyngeal cancer. *Nat Genet* 2016; **48:** 1544-1550. 10.1038/ng.3685 PMC5131845 27749845

[44] Shete S, Liu H, Wang J *et al.* A Genome-Wide Association Study Identifies Two Novel Susceptible Regions for Squamous Cell Carcinoma of the Head and Neck. *Cancer Res* 2020; **80:** 2451-2460. 10.1158/0008-5472.CAN-19-2360 PMC7299763 32276964

[45] Boccia S, Hashibe M, Gallì P *et al.* Aldehyde dehydrogenase 2 and head and neck cancer: a meta-analysis implementing a Mendelian randomization approach. *Cancer Epidemiol Biomarkers Prev* 2009; **18:** 248-254. 10.1158/1055-9965.EPI-08-0462 19124505

[46] Wei S, Liu Z, Zhao H *et al.* A single nucleotide polymorphism in the alcohol dehydrogenase 7 gene (alanine to glycine substitution at amino acid 92) is associated with the risk of squamous cell carcinoma of the head and neck. *Cancer* 2010; **116:** 2984-2992. 10.1002/cncr.25058 PMC2891145 20336794

[47] Wyss A, Hashibe M, Chuang S-C *et al.* Cigarette, cigar, and pipe smoking and the risk of head and neck cancers: pooled analysis in the International Head and Neck Cancer Epidemiology Consortium. *Am J Epidemiol* 2013; **178:** 679-690. 10.1093/aje/kwt029 PMC3755640 23817919

[48] Lee Y-C A, Boffetta P, Sturgis E M *et al.* Involuntary smoking and head and neck cancer risk: pooled analysis in the International Head and Neck Cancer Epidemiology Consortium. *Cancer Epidemiol Biomarkers Prev* 2008; **17:** 1974-1981. 10.1158/1055-9965.EPI-08-0047 PMC2561190 18708387

[49] Wyss A B, Hashibe M, Lee Y-C A *et al.* Smokeless Tobacco Use and the Risk of Head and Neck Cancer: Pooled Analysis of US Studies in the INHANCE Consortium. *Am J Epidemiol* 2016; **184:** 703-716. 10.1093/aje/kww075 PMC5141945 27744388

[50] Asthana S, Labani S, Kailash U, Sinha D N, Mehrotra R. Association of Smokeless Tobacco Use and Oral Cancer: A Systematic Global Review and Meta-Analysis. *Nicotine Tob Res* 2019; **21:** 1162-1171. 10.1093/ntr/nty074 29790998

[51] Purdue M P, Hashibe M, Berthiller J *et al.* Type of alcoholic beverage and risk of head and neck cancer - a pooled analysis within the INHANCE Consortium. *Am J Epidemiol* 2009; **169:** 132-142. 10.1093/aje/kwn306 PMC2727255 19064644

[52] Voltzke K J, Lee Y-C A, Zhang Z-F *et al.* Racial differences in the relationship between tobacco, alcohol, and the risk of head and neck cancer: pooled analysis of US studies in the INHANCE Consortium. *Cancer Causes Control* 2018; **29:** 619-630. 10.1007/s10552-018-1026-z PMC6626318 29761303

[53] Berthiller J, Lee Y-C A, Boffetta P *et al.* Marijuana smoking and the risk of head and neck cancer: pooled analysis in the INHANCE consortium. *Cancer Epidemiol Biomarkers Prev* 2009; **18:** 1544-1551. 10.1158/1055-9965.EPI-08-0845 PMC3046921 19423532

[54] Stott-Miller M, Chen C, Chuang S-C *et al.* History of diabetes and risk of head and neck cancer: a pooled analysis from the international head and neck cancer epidemiology consortium. *Cancer Epidemiol Biomarkers Prev* 2012; **21:** 294-304. 10.1158/1055-9965.EPI-11-0590 PMC3275674 22144496

[55] Negri E, Boffetta P, Berthiller J *et al.* Family history of cancer: pooled analysis in the International Head and Neck Cancer Epidemiology Consortium. *Int J Cancer* 2009; **124:** 394-401. 10.1002/ijc.23848 PMC3711193 18814262

[56] Khetan P, Boffetta P, Luce D *et al.* Occupations and the Risk of Head and Neck Cancer: A Pooled Analysis of the International Head and Neck Cancer Epidemiology (INHANCE) Consortium. *J Occup Environ Med* 2019; **61:** 397-404. 10.1097/JOM.0000000000001563 PMC6613803 31268937

[57] Conway D I, Brenner D R, McMahon A D *et al.* Estimating and explaining the effect of education and income on head and neck cancer risk: INHANCE consortium pooled analysis of 31 case-control studies from 27 countries. *Int J Cancer* 2015; **136:** 1125-1139. 10.1002/ijc.29063 PMC4531373 24996155

[58] Conway D I, Hovanec J, Ahrens W *et al.* Occupational socioeconomic risk associations for head and neck cancer in Europe and South America: individual participant data analysis of pooled case-control studies within the INHANCE Consortium. *J Epidemiol Community Health* 2021; **75:** 779-787. 10.1136/jech-2020-214913 PMC8292575 33622804

